# Non-Coding RNAs as Predictive Biomarkers to Current Treatment in Metastatic Colorectal Cancer

**DOI:** 10.3390/ijms18071547

**Published:** 2017-07-17

**Authors:** Ingrid Garajová, Manuela Ferracin, Elisa Porcellini, Andrea Palloni, Francesca Abbati, Guido Biasco, Giovanni Brandi

**Affiliations:** 1 Department of Experimental, Diagnostic and Specialty Medicine (DIMES), Sant’Orsola-Malpighi Hospital, University of Bologna, 40138 Bologna, Italy; manuela.ferracin@unibo.it (M.F.); elisa.porcellini3@unibo.it (E.P.); andrepalloni@gmail.com (A.P.); francesca.abbati1989@gmail.com (F.A.); guido.biasco@unibo.it (G.B.); giovanni.brandi@unibo.it (G.B.); 2 Interdepartmental Centre of Cancer Research “Giorgio Prodi”, University of Bologna, 40138 Bologna, Italy

**Keywords:** colorectal cancer, non-coding RNA, microRNA, predictive biomarker

## Abstract

The onset and selection of resistant clones during cancer treatment with chemotherapy or targeted therapy is a major issue in the clinical management of metastatic colorectal cancer patients. It is possible that a more personalized treatment selection, using reliable response-to-therapy predictive biomarkers, could lead to an improvement in the success rate of the proposed therapies. Although the process of biomarker selection and validation could be a long one, requiring solid statistics, large cohorts and multicentric validations, non-coding RNAs (ncRNAs) and in particular microRNAs, proved to be extremely promising in this field. Here we summarize some of the main studies correlating specific ncRNAs with sensitivity/resistance to chemotherapy, anti-VEGF therapy, anti-EGFR therapy and immunotherapy in colorectal cancer (CRC).

## 1. Introduction

Colorectal cancer (CRC) is the third most common cancer in western countries [1]. In recent decades, important treatment improvements in the field of CRC have been achieved. This is especially due to the incorporation of monoclonal antibodies against vascular endothelial growth factor (VEGF) and epidermal growth factor receptor (EGFR) in the treatment of metastatic CRC [2]. The efficacy of anti-EGFR antibodies depends on the presence of RAS/BRAF mutations and moreover, the efficacy of both anti-EGFR and anti-VEGF antibodies seems to vary between the right-sided (RS) and left-sided (LS) colon tumors [3,4]. In addition, the right sequence of the treatments is still under debate. The overall survival (OS) of patients with metastatic CRC is currently around 30 months, which is almost doubled when compared to 5-FU monotherapy era [5,6].

A fraction of patients treated with conventional cytostatics with/or anti-VEGF/anti-EGFR therapies do not respond to therapy. Therefore, predictive biomarkers for effectiveness of chemotherapy and anti-VEGF/anti-EGFR are necessary. The identification of biomarkers for treatment efficacy might lead to more personalized, more effective and less toxic treatments for CRC patients.

Within this context, a promising source of response-to-therapy biomarkers is constituted by non-coding RNAs (ncRNAs). It is a class of regulatory RNAs whose main members could be either small or long ncRNAs. Among the small ncRNAs, microRNAs (miRNAs) have been extensively studied as cancer biomarkers due to their stability in formalin-fixed paraffin embedded (FFPE) tissues and in the blood circulation. Recently, the role of long ncRNAs (lncRNA) in tumorigenesis and response to therapy has started to be investigated.

## 2. Non-Coding RNAs as Predictive Biomarkers to Chemotherapy in Colorectal Cancer

Several studies have reported the possible use of ncRNAs as predictive biomarkers to anticancer treatments in CRC patients. Salendo et al. performed a genome-wide miRNA profiling in twelve CRC lines and established individual in vitro signature of chemosensibility [7]. The authors further analyzed the selected miRNAs in pre-therapeutic biopsies from rectal cancer patients who underwent neoadjuvant (preoperative) chemoradiation. In particular, only the overexpression of let-7 was associated with a good prognosis in 128 rectal cancer patients (*p* = 0.028), and therefore might serve as predictive biomarker in this setting [7]. In another study, forced expression of miR-21 significantly inhibited apoptosis and increased cell growth, invasion and resistance of tumor cells to chemotherapeutic agent 5-FU and radiation [8]. Moreover, miR-21 might indirectly regulate the expression of thymidine phosphorylase (TP) and dihydropyrimidine dehydrogenase (DPD), enzymes involved in metabolism of fluoropyrimidines [8]. Valeri et al. demonstrated that miR-21 downregulates human mutS homolog 2 (hMSH2) and 6 (hMSH6), which are the core mismatch repair (MMR) proteins. Furthermore, xenograft studies confirmed that miR-21 overexpression reduces the therapeutic efficacy of 5-FU. These findings suggest that silencing miR-21 could restore the sensibility of CRC cells to 5-FU [9]. Moreover, downregulation of other miRNAs was associated with chemoresistance. Karaayvaz et al. showed in 22 pair of fresh-frozen controls and CRC patients that miR-129 level was significantly decreased in tumor tissues compared with normal controls (*p* < 0.0001) and in another set of 61 FFPE CRCs that the expression of miR-129 was significantly reduced in patients with stage 3 and stage 4 disease compared with both normal or adenoma tissues (*p* < 0.0001) [8].

This miRNA increased the cytotoxic effect of 5-FU both in vitro and in vivo and suggest a potential of developing miR-129-based therapeutic strategies to enhance or overcome the resistance to 5-FU in CRC [10]. Dong et al. observed that miR-429 expression is correlated with increased malignant potential, poor prognosis and chemosensitivity. In particular, miR-429 expression was significantly increased in CRC primary tissue compared with adjacent normal tissue (*p* < 0.0001) and in CRC patients’ serum vs. healthy subjects (*p* < 0.0001). In addition, miR-429 expression was positively correlated with TNM stage, lymph node metastasis, distant metastasis and tumor size (*p* = 0.00001, *p* = 0.0001, *p* = 0.00001 and *p* = 0.036 respectively). Kaplan–Meier analysis on these patients indicated that subjects with high expression of miR-429 were associated with shorter overall survival compared with the low expressing CRC patients (*p* = 0.0021). Moreover, miR-429 overexpression was associated with poor response to 5-FU-based chemotherapy in patients with CRC (*p* < 0.001) [11]. Another study observed that 5-FU-resistant CRC cells show elevated levels of miR-577. Heat shock protein 27 (HSP27) was identified as a target gene of miR-577. Interestingly, enforced expression of HSP27 modulated the effects of miR-577 on 5-FU sensitivity in CRC cells [12]. A study of Xu et al. observed that miR-1915 could play a role in the development of multidrug resistance in CRC cells by modulation of apoptosis though Bcl-2 [13]. The authors found that increased levels of miR-1915 in the mimics-transfected HCT116/L-OHP cells decreased Bcl-2 protein level and sensitized CRC cells to several anticancer drugs. In another study, it was found that miR-122 was differently expressed between 5-FU resistant and sensitive CRC cells. Overexpression of miR-122 in 5-FU-resistant cells resensitizes 5-FU resistance through the inhibition of glycolytic enzyme pyruvate kinase type M2 (PKM2) both in vitro and in vivo [14]. To et al. investigated the expression of an efflux transporter, ABCG2, in CRC and normal colonic mucosa [15]. In this study, conducted on 12 CRC patients and 2 polyp samples, miR-203 was found to be downregulated in CRC specimens in comparison to normal colon mucosa. Downregulation of miR-203 induced ABCG2 promoter methylation, through its target DNA methyltransferase DNMT3b activation, and a significant reduction in ABCG2 expression. CRC tumors have a significantly lower ABCG2 expression than the adjacent normal colon tissues. Downregulation of miR-203 in CRC caused ABCG2 promoter methylation and significantly lower ABCG2 expression in CRC. Therefore, the restoration of ABCG2 function via modulating this new miRNA-methylation mechanism might represent an attractive strategy in CRC prevention and cure [8]. Lopes-Ramos et al. analyzed miRNA expression profile in rectal tumor biopsies prior to neoadjuvant chemoradiotherapy (nCRT) and found four miRNAs differentially expressed in patients who obtained complete or incomplete response (*p* < 0.0001): in particular three miRNAs were overexpressed in complete responders (miR-21-5p, miR-1246, and miR-1290-3p) and one, miR-205-5p, was overexpressed in incomplete responders [16]. Moreover, in rectal cancer patients who obtained the complete response, overexpression of miR-21-5p was observed. Interestingly, in a subset of patients with complete response after nCRT with early local recurrence was characterized by downregulation of miR-21-5p, similar to that in incomplete responders [16].

The same setting of patients affected by rectal cancer who underwent the nCRT were analyzed also by Caramés et al. [17]. The authors found that the majority of pre-treatment biopsies (78% of cases) are characterized by miR-21 overexpression, which also correlate with a worse response after nCRT (*p* = 0.013). Sensitivity, specificity, negative predictive values, and positive predictive value were 86.6%, 60%, 42.8%, and 92%, respectively. Moreover, multivariate analysis confirmed the clinical significance of miR-21 in determining nCRT response in locally advanced rectal cancer patients [17]. Another study showed the possible correlation between miRNAs and oxaliplatin resistance in CRC patients. Overexpression of miR-153 was correlated with advanced CRC stage. Moreover, upregulation of miR-153 was observed to promote CRC invasiveness indirectly by inducing matrix metalloprotease enzyme 9 production and resistance to oxaliplatin and cisplatin directly by inhibiting the Forkhead transcription factor Forkhead box O3a (FOXO3a), both in vitro and in vivo [18]. The discovery that ncRNAs are released from tumor cells and can be retrieved in the blood, either as cell-free molecules or inside microvescicles, opened novel possibilities for biomarker identification. Cell-free microRNAs are extremely stable in serum and plasma and both tissues can be easily collected from patients. Recently, miRNA levels have been correlated with response to therapy in several cancer types, including colorectal cancer [19]. Chen et al. evaluated the expression of serum miR-19a in CRC patients treated with FOLFOX chemotherapy regimen [20]. In particular, miR-19a seemed to be significantly up-regulated in patients who were resistant to FOLFOX regimen, suggesting its potential as an easily assessable biomarker for predicting and monitoring resistance to FOLFOX chemotherapy (AUC 0.679, 95% confidence interval [CI] 0.534–0.824, with a sensitivity of 66.7% and specificity of 63.9%) Circulating miR-19a was upregulated. Kjersem et al. identified three circulating miRNAs in plasma of CRC patients (miR-106a, miR-130b, and miR-484), whose upregulation was correlated with resistance to FOLFOX regimen (*p* < 0.05 for each miRNA) [21]. In another study, Zhang et al. performed a global analysis of miRNA expression in the serum of 250 CRC patients treated with chemotherapy [22] and were able to identify and validate a 5-miRNA signature predictive of chemotherapy sensitivity with an AUC of 0.84–0.91 in two independent validation cohorts.

Among the lncRNAs involved in chemotherapy resistance, TUG1 has been recently proposed as a mediator of methotrexate resistance through its upregulation in methotrexate-resistant CRC and sponge activity on miR-186 [23]. Li et al. investigated in serum samples and tissue from patients with metastatic colorectal cancer, the role of the lncRNA MALAT1 in oxaliplatin resistance [24]. High expression of MALAT1 in CRC patients was associated with poor prognosis (*p* = 0.003) and chemoresistance to FOLFOX treatment, through a mechanism related to E-cadherine downregulation. In addition, a study by Bian et al. described a regulatory network involving the lncRNA UCA1, miR-204-5p and the transcription factor CREB1 in 5-FU sensitivity of CRC cells [25].

Moreover, also LINE-1 (long interspersed nuclear elements-1) has been proposed as biomarker for colon cancer being its hypomethylation associated with a worse prognosis [26].

Recently, several studies showed that patients, with a hypomethylated LINE-1 and treated with FOLFOX had a greater risk of early postoperative recurrence, poor diagnosis and a shorter period of disease-free survival in Stage III [27,28].

In conclusion, miRNAs and lncRNAs possess high potential as predictive markers for therapeutic response to chemotherapeutic drugs and could be evaluated as therapeutic targets in CRC.

## 3. Non-Coding RNA as Predictive Biomarkers for Anti-VEGF Monoclonal Antibodies in Colorectal Cancer

VEGF-neutralizing therapy with bevacizumab has become a standard of care for metastatic CRC in the first and second-line of treatment. Hansen et al. investigated the predictive value of circulating plasma miR-126 in sixty-eight patients with metastatic CRC that were treated with first-line chemotherapy combined with bevacizumab [29]. Plasma samples were collected before the therapy initiation, after three weeks and at the time of radiologic progression according to RECIST criteria. The authors observed that changes in circulating miR-126 during the treatment correlate with response/resistance to the treatment with chemotherapy and bevacizumab. Specifically, cell-free miR-126 level was significantly higher in non-responding patients than in stable or responding patients (*p* < 0.05), both at the first clinical evaluation and at progression. Therefore, circulating miR-126 might present a new biomarker for anti-angiogenic containing regimes [29]. The role of miR-126 in angiogenesis was described in the literature also by other authors. Chistiakov et al. described the role for miR-126 as master regulator of embryonic vasculogenesis or Du et al. demonstrated that miR-126 deregulation contributed to neovascularization in HCC [30,31]. In another study, expression levels of miRNAs were analyzed in cancer tissue in order to predict effectiveness of first-line chemotherapy regimen (capecitabine and oxaliplatin) and bevacizumab in patients with metastatic CRC. miRNA profiling of a total of 754 miRNAs were analyzed. Overexpression of miR-664-3p and downregulation of miR-455-5p were predictive of improved outcome chemotherapy and bevacizumab regimen (*p* = 0.02 and *p* = 0.02, respectively) [32]. Recently, a correlation between regorafenib and miRNAs has been described. Regorafenib is a multi-kinase inhibitor used in metastatic colorectal cancer that blocks a series of protein kinases involved in angiogenesis [(VEGF receptors 1-3 and tyrosine receptor kinase-2 (TIE2)], oncogenesis (KIT, RET, RAF1, BRAF and BRAF V600E) and the tumor microenvironment [platelet-derived growth factor receptor (PDGFR) and fibroblast growth factor receptor (FGFR)] [33]. Chen et al. described that regorafenib can directly bind to miR-21 pre-element, thus preventing RNase dicer-meditated cleavage of the pre-element to mature miR-21 [34].

## 4. Non-Coding RNA as Predictive Biomarkers Anti-EGFR Monoclonal Antibodies in Colorectal Cancer

Anti-EGFR antibodies, such as cetuximab and panitumumab, have been approved and routinely used for the treatment of metastatic CRC. Wild-type (wt) KRAS and NRAS status is required for anti-EGFR treatment initiation. Several miRNAs, including miR-143, miR-145, let-7 and miR-18a∗, act as tumor suppressors and repress the expression of KRAS [35] and vice-versa, KRAS regulates the expression of different oncogenic miRNAs, including miR-200c, miR-221/222, miR-210 and miR-181∗ [36]. Cappuzzo et al. investigated the role of miRNAs as potentional predictive biomarkers to anti-EGFR monoclononal antibodies in metastatic CRC patients. The authors identified three miRNAs (let-7c/miR-99a/miR-125b) as a signature that could predictably improve response in wtKRAS metastatic CRC patients treated with EGFR target-therapies [37]. The patients with high-intensity signatures had a significantly longer PFS (6.1 vs. 2.3 months; *p* = 0.02) and OS (29.8 vs. 7.0 months, *p* = 0.08) than patients with low-intensity signatures. In the validation cohort, patients with high signature had significantly longer PFS and OS than individuals with low-intensity signatures (PFS 7.8 vs. 4.3 months, *p* = 0.02; OS 12.8 vs. 7.5 months, *p* = 0.02). The potential role of miRNAs as predictive biomarkers to anti-EGFR therapy was investigated also by Lupini et al. [38]. First, miRNA profiling was performed on the exploratory cohort of CRC patients treated with cetuximab. The authors identified nine miRNAs with significantly different expression between cetuximab therapy responders and non-responders. Subsequently, the validation was performed on two independent patients’ groups treated with cetuximab or panitumumab. Expression of miR-31-3p and miR-31-5p were found to correlate with outcome of metastatic CRC patients treated with cetuximab but not panitumumab [38,39]. This was confirmed also by a study of Igarashi et al. who confirmed that miR-31-5p overexpression is associated with shorter progression-free survival in patients with CRC treated with anti-EGFR therapeutics (*p* = 0.003) [40]. Downregulation of miR-181a was associated with poor survival in patients with CRC (*p* = 0.019) and furthermore, miR-181a expression might predict progression-free survival in EGFR-targeted therapy [39]. These two anti-EGFR antibodies can modulate cellular immunity of CRC cells. Moreover, anti-tumor immune responses of antibody and EGFR signaling pathway were shown to be regulated by miRNAs [41,42]. miR-143 and miR-145 are downregulated in CRC. Gomes et al. evaluated cetuximab-mediated antibody-dependent cellular cytotoxicity (ADCC). Overexpression of miR-143 or miR-145 enhanced cell sensitivity to cetuximab, resulting in a significant increase of cetuximab-mediated ADCC. The authors conclude that reintroduction of miR-143 or miR-145 might lead to resensitize CRC cells to cetuximab by stimulating cetuximab-dependent ADCC to induce cell apoptosis [43]. The possible interactions among miRNAs and anti-EGFR therapy was revealed also by Zhou et al. In this study, combination treatment with miR-133b mimics and cetuximab, exhibited improved inhibitory effects on the growth and invasion of CRC cells compared with treatment with either alone [44].

## 5. Non-Coding RNA as Predictive Biomarkers Immunotherapy in Colorectal Cancer

Immune checkpoint molecules including programmed cell death 1 (PD-1) and its ligand (PD-L1), cytotoxic T-lymphocyte-associated protein 4 (CTLA-4), lymphocyte activation gene (LAG-3), and indoleamine 2,3-dioxygenase (IDO) have been proposed as possible targets for immunotherapy in CRC [45]. The clinical activity of immunotherapy has been investigated in several clinical trials. A phase 2 study with pembrolizumab, an anti-PD-1 antibody, showed a clinical activity in MSI-H CRC. The immune-related objective response rate and immune-related PFS rate were 40% and 78%, respectively, for MSI-H CRCs and 0% and 11% in MSS CRCs [46,47]. Another phase 2 trial investigated the efficacy of nivolumab (anti-PD-1) and ipilimumab (anti-CTLA-4) in MSI-H mCRC. Partial remission and stable disease was seen in 27% and 24% in nivolumab arm and 15% and 65% in nivolumab and ipilimumab arm. Median PFS and OS are available only in nivolumab arm and were 5.3 months and 16.3 months, respectively. Interestingly, clinical activity and safety of atezolizumab (anti-PD-L1) and cobimetinib (MEK1/2 inhibitor) was investigated in CRC patients, independently to microsatellite stability status. In the KRAS mutant CRC cohort, partial remission were seen in 20% of patients and stability in 20% patients, 50% of the patients showed progression disease and in another 10% the response was not possible [48].

Some authors suggest the association between PD-L1 expression and mutational status of BRAF and KRAS in CRC. In particular, PD-L1 overexpression was associated with lower frequency of KRAS and more frequent BRAF mutations [49]. Moreover, PD-L1 is a target of several microRNAs and targeting PD-L1 by administering anti-miRNA therapy might potentially be a clinically effective therapeutic strategy in metastatic CRC [50]. It has been demonstrated that PTEN loss increases PD-L1 protein expression [51], as well as miR-21, miR-20b and miR-130b overexpression [52]. Furthermore, miR-484 is significantly decreased in MSI CRC and functions as a tumor suppressor to inhibit MSI CRC cell viability [42]. Mima et al. suggest a possible role of miRNA deregulation in suppressing antitumor T-cell–mediated adaptive immune response. The authors identify miR-21 as inversely correlated with the presence of memory T-cells in tumor microenvironment suggesting miR-21 as a potential target for immunotherapy in CRC [53].

## 6. Conclusions and Future Directions

In conclusion, several studies have demonstrated the importance of miRNAs and lncRNAs as biomarkers to predict the efficacy of conventional chemotherapy, anti-VEGF and anti-EGFR targeted therapies in CRC, as summarized in Table 1. Moreover, miRNAs are critical regulator of immune response [53] and therefore a role as response predictors to immunotherapy in CRC could be expected. Due to their stability in FFPE tissues, prognostic or predictive microRNAs can be quantified using diagnostic biopsies as a source of cancer material; therefore the analysis could be easily performed during the standard diagnostics procedures.

Recently, ncRNA detection in patients’ body fluids (e.g., serum and plasma) proved to be a minimally invasive source of prognostic biomarkers. The opportunity to monitor specific ncRNA levels in serum or plasma in order to get an insight into the responsiveness of tumor cells, before starting a new therapeutic regimen or when the therapy is ongoing, is extremely appealing. In addition, their quantification could be easily implemented in liquid biopsy procedures. However, large, multicentric studies validating the most promising candidates are still lacking in this field for metastatic CRC, and standardized protocols for circulating ncRNA quantification are still under development. Nonetheless, we think that in the next future the periodic assessment of specific ncRNAs in the blood of treated CRC patients could be valuable if integrated with ctDNA mutational analysis or considered as an alternative when ctDNA is not informative.

## Figures and Tables

**Table 1:** Summary of ncRNAs that are response-to-therapy predictors in CRC.

Prognostic ncRNA *	Tissue	Effect	Ref.
Conventional cytostatics			
miR-21-5p	Tumor	Upregulation increases resistance to 5-FU	[7,8]
miR-129-5p	Tumor	Downregulation increases resistance to 5-FU	[10]
miR-429	Tumor	Upregulation increases resistance to 5-FU	[11]
miR-577	Tumor	Upregulation increases resistance to 5-FU	[12]
miR-1915-3p	Tumor	Downregulation increases resistance to chemotherapy	[13]
miR-122-5p	Tumor	Downregulation increases resistance to 5-FU	[14]
miR-21-5p	Tumor	Upregulation decreases resistance to nCRT	[16]
miR-153-3p	Tumor	Upregulation increases resistance to oxaliplatin	[18]
miR-19a-3p	Tumor	Upregulation increases resistance to FOLFOX therapy	[34]
miR-106a-5p, miR-130b-3p, miR-484	Plasma/serum	Upregulation increases resistance to FOLFOX therapy	[21]
miR-20a-5p, miR-130, miR-145-5p, miR-216a-5p and miR-372-3p	Plasma/serum	Response prediction to 5-FU-based adjuvant therapy	[22]
TUG1	Tumor	Upregulation increases resistance to methotrexate	[23]
MALAT1	Tumor	Upregulation increases resistance to FOLFOX	[24]
UCA1	Tumor	Upregulation increases resistance to 5-FU	[25]
Anti-VEGF antibodies			
miR-126-3p	Plasma/serum	Upregulation increases resistance to CT and bevacizumab	[29]
miR-664-3p	Tumor	Upregulation increases sensitivity to CT and bevacizumab	[32]
miR-455-5p	Tumor	Downregulation increases sensitivity to CT and bevacizumab	[32]
Anti-EGFR antibodies			
miR-99a-5p/let-7c/miR-125b-5p	Tumor	Response prediction to EGFR target-therapies	[37]
miR-31-3p and miR-31-5p	Tumor	Response prediction to cetuximab but not panitumumab	[38,39]
miR-31-5p	Tumor	Upregulation increases resistance to EGFR target-therapies	[40]
miR-181a-5p	Tumor	Downregulation increases resistance to EGFR target-therapies	[39]
miR-143-3p	Tumor	Upregulation increases sensitivity to cetuximab	[43]
miR-145-5p	Tumor	Upregulation increases sensitivity to cetuximab	[43]

* The nomenclature of mature miRNAs has been updated to the latest miRBase release (v.21).
